# Peripheral artery disease assessed by ankle-brachial index in patients with established cardiovascular disease or at least one risk factor for atherothrombosis - CAREFUL Study: A national, multi-center, cross-sectional observational study

**DOI:** 10.1186/1471-2261-11-4

**Published:** 2011-01-19

**Authors:** Ahmet K Bozkurt, Ilker Tasci, Omur Tabak, Mehmet Gumus, Yesim Kaplan

**Affiliations:** 1 Department of Cardiovascular Surgery, Istanbul University Cerrahpasa Medical School, Istanbul, Turkey; 2 Department of Internal Medicine, Gulhane School of Medicine, Ankara, Turkey; 3 Internal Medicine Clinic, Ministry of Health Istanbul Training and Research Hospital, Istanbul, Turkey; 4 Avcılar Anadolu Hospital, Istanbul, Turkey; 5 sanofi aventis, Istanbul, Turkey

## Abstract

**Background:**

To investigate the presence of peripheral artery disease (PAD) via the ankle brachial index (ABI) in patients with known cardiovascular and/or cerebrovascular diseases or with at least one risk factor for atherothrombosis.

**Methods:**

Patients with a history of atherothrombotic events, or aged 50-69 years with at least one cardiovascular risk factor, or > = 70 years of age were included in this multicenter, cross-sectional, non-interventional study (DIREGL04074). Demographics, medical history, physical examination findings, and physician awareness of PAD were analyzed. The number of patients with low ABI (< = 0.90) was analyzed.

**Results:**

A total of 530 patients (mean age, 63.4 ± 8.7 years; 50.2% female) were enrolled. Hypertension and dyslipidemia were present in 88.7% and 65.5% of patients, respectively. PAD-related symptoms were evident in about one-third of the patients, and at least one of the pedal pulses was negative in 6.5% of patients. The frequency of low ABI was 20.0% in the whole study population and 30% for patients older than 70 years. Older age, greater number of total risk factors, and presence of PAD-related physical findings were associated with increased likelihood of low ABI (*p *< 0.001). There was no gender difference in the prevalence of low ABI, PAD symptoms, or total number of risk factors. Exercise (33.6%) was the most common non-pharmacological option recommended by physicians, and acetylsalicylic acid (ASA) (45.4%) was the most frequently prescribed medication for PAD.

**Conclusion:**

Our results indicate that advanced age, greater number of total risk factors and presence of PAD-related physical findings were associated with increased likelihood of low ABI. These findings are similar to those reported in similar studies of different populations, and document a fairly high prevalence of PAD in a Mediterranean country.

## Background

Previous studies have consistently documented that peripheral artery disease (PAD) is a significant predictor of future cardiovascular events, such as myocardial infarction or stroke [1-3]. Epidemiological studies have estimated that the prevalence of PAD in the general population is up to 10%, and is twice as high in people older than 70 years [4,5]. Moreover, for each patient with symptomatic PAD, there are approximately three to four undiagnosed subjects with asymptomatic PAD [6].

Both symptomatic and asymptomatic PAD have been found to be associated with increased risk of cardiovascular disease (CVD) and adverse risk profiles [5,7,8]. The Reduction of Atherothrombosis for Continued Health (REACH) registry indicated that 60% of patients with PAD had atherosclerotic disease in other vascular beds [9]. PAD patients were reported to have a 6.6-fold greater risk of death from coronary heart disease (CHD) than patients without PAD, and a 3.1-fold greater mortality from any cause [10,11]. Cross-sectional studies showed that almost half of all PAD subjects seemed to have some clinical evidence of CHD or cerebrovascular disease [12]. However, due to the silent nature of this disease and the subtle findings on physical examination [11,13], PAD has been considered the least effectively managed major atherosclerotic vascular disorder [5,9]. Although PAD is a strong prognostic marker for future cardiovascular events, previous research indicates that only a small fraction of patients with PAD are properly diagnosed [14].

The ankle brachial index (ABI) is a sensitive and cost-effective screening tool for PAD. In addition to its value in the diagnosis of PAD, the ABI can also be used to assess generalized atherosclerosis [15]. Previous studies have shown that a low ABI (≤ 0.90) has a specificity of greater than 98% for the diagnosis of PAD and a specificity of 92% for the prediction of CHD and stroke [16-18]. Among the elderly, an ABI of 0.90 or less indicates subclinical atherosclerosis and is a strong predictor of total mortality and cardiovascular morbidity and death in those with no prior history of clinical cardiovascular disease [19].

The primary aim of the present study was to investigate the use of low ABI (≤ 0.90) as an indicator of PAD in a target population admitted to internal medicine outpatient clinics in Turkey. Our secondary objectives were to evaluate the characteristics of individuals with low ABIs with respect to gender and the presence of established CVD and/or major cardiovascular risk factors, and to assess physician awareness of PAD management.

## Methods

### Study population and objectives

The CAREFUL study enrolled patients admitted to internal medicine outpatient clinics in Turkey from February 23 to June 19, 2009. In this national, multi-center, cross-sectional, and observational study (DIREGL04074), individuals from 27 centers, representative of the geographical composition of the whole country, were examined. To assure that our study population was representative of Turkey as a whole, participating centers were selected by the Project Advisory Board using stratified selection based on geography and type of institution among all internal medicine clinics in Turkey, and on the distribution of investigators in relation to type of institutions where they worked (university hospital, state hospital, or private hospital).

### Inclusion and exclusion criteria

The main inclusion criteria were age of 50-69 years and presence of established CVD or at least one major cardiovascular risk factor (smoking, diabetes, hypertension, and dyslipidemia, as defined by the TASC II report [20]). The exclusion criteria were presence of previously diagnosed PAD, refusal to participate, and conditions that required hospitalization, such as cancer or a life threatening illness.

### Working protocol

The CAREFUL study was approved by the local ethics committee prior to initiation, and all participants provided written informed consent following a detailed explanation of the objectives and protocol. During the study period of 4 months, in each center, the first patient of each investigator's working day who fulfilled the inclusion criteria was invited to participate; if this patient refused, then the next patient was asked. This continued until there were 20 patients recruited from each center. Each patient was examined at a single visit, and there were no interventions involving ongoing medications and/or decisions of the physicians. Patient demographics, history of atherothrombotic events, smoking status, presence of hypertension, diabetes mellitus (DM), dyslipidemia (risk factors for PAD), and medications in the last 6 months (including antihypertensives, anti-diabetics, lipid-lowering drugs, and antithrombotic agents) were recorded during this visit. Blood pressure, heart rate, body weight, height, body mass index (BMI), waist circumference, and physical examination findings specific to PAD (absence of pedal pulses, arterial murmur, and trophic changes of the foot) were also recorded.

For patients diagnosed with PAD, the recommended treatment strategy was also noted. A three-item modified Edinburgh Claudication Questionnaire on PAD symptoms was given to all patients and an eight-item questionnaire on awareness of PAD was given to the physicians [21]. The PAD awareness questionnaire included questions about the age group of patients who should be considered for measurement of ABI, evaluation of obese/diabetic/actively smoking patients by physical examination for ABI with the possibility of PAD, the objectives of PAD treatment (according to Turkish Society of Cardiovascular Surgery), the role of medical history and physical examination in the diagnosis of PAD, the diagnostic option(s) for patients who required further investigation, and the "treatment of choice" for ischemic events.

### Measurement of ankle brachial index

Measurement of systolic pressures of the four limbs was performed in all patients using a standard sphygmomanometer in a quiet room, with the patient supine for at least 5 min before measurement. Right and left arm and ankle (posterior tibial artery and dorsalis pedis artery) systolic blood pressures were measured by trained physicians using a handheld Doppler ultrasound (Vascular Doppler 8 MHz, Hedeco, Japan) and a manually operated blood pressure cuff with a 15 cm-wide bladder. A cycle of measurements (right arm, right ankle, left ankle, left arm) was repeated, and the means of two measurements for each limb were used to calculate the ABI. Finally, the ratio of the highest systolic pressure in the ankle to the higher of the left or right brachial systolic pressure was used to define the ABI [15,22]. An ABI of 0.90 or less was considered abnormal and indicative of PAD [11].

### Statistical analyses

The AGATHA study reported that 30.9% of patients with cardiovascular risk factors had ABIs of 0.90 or less and that 27.6% of these patients had involvement in both arterial beds [23]. Thus, based on the estimation of the rate of an event (ABI ≤ 0.90) of 30%, an error of 6%, a 95% confidence interval (CI), α of 0.05, and power of 80%, the sample size was calculated as 437. Assuming a missing data probability of 10-15%, we enrolled ~500 patients. Statistical analysis was performed with SPSS version 13.0 (SPSS Inc. Chicago, IL, USA). Chi-square (χ^2^) and Fischer's exact tests were used for comparisons of categorical data. Data are expressed as means ± standard deviations (SDs) or as percent (%) and 95% CI where appropriate. A *p*-value less than 0.05 was considered statistically significant.

## Results

### Demographics of the study population

A total of 533 patients underwent ABI measurement for this study. Due to inappropriate enrollment, three participants from different centers were excluded prior to analysis. Among the 530 enrolled patients, 264 were male (49.8%) and 266 were female. Mean age was 63.4 ± 8.7 years in the entire group, and was 63.2 ± 8.5 years (range: 50-84) for men and 63.8 ± 8.9 years (range 50-88) for women. Among men, 39% were 50-59 years of age, 37.9% were 60-69, 20.1% were 70-79, and 3% were 80-89 years. Among women, 37.2% were 50-59 years of age, 39.5% were 60-69, 16.2% were 70-79, and 7.1% were 80-89 years. Gender distributions of the different age groups were similar.

### Presence of cardiovascular disease or risk factors and concomitant drug use

A total of 27.9% of patients had histories of CHD and 7.4% had histories of cerebrovascular disease. CHD was more prevalent in men than in women (33.3% for men *vs. *22.6% for women, *p *= 0.006), but history of cerebrovascular disease was similar in both genders (7.6% for men *vs. *7.1% for women, *p *= 0.848). Regarding risk factors, 42.8% of participants identified themselves as active or past smokers, 88.7% were hypertensive or were treated for this condition, 65.5% had dyslipidemia or were treated for this condition, and 59.4% had type II diabetes mellitus (DM) or were treated for this condition.

Table 1 provides the gender distribution, clinical background, and vital signs for all enrolled patients. Females were more likely to have DM and be nonsmokers than males (*p *< 0.01 and *p *< 0.001, respectively; Table 1). There were no gender differences in blood pressure, heart rate, BMI, or waist circumference (Table 1). Among all 530 enrolled patients, 58 (10.9%) had one, 186 (35.1%) had two, 215 (40.6%) had three, and 71 (13.4%) had four major cardiovascular risk factors. There was no gender difference in the number of accompanying risk factors.

**Table 1:** Gender-based distribution clinical background and vital signs of the study population

	Male (n = 264)	Female (n = 266)
Past history of coronary heart disease	**n(%)**	
Overall	88(33.3)*	60(22.6)
Stable angina pectoris	22(25.0)	23(38.3)
Unstable angina pectoris	7(8.0)	4(6.7)
Coronary bypass surgery	30(34.1)	13(21.7)
Percutaneous coronary intervention	25(28.4)	13(21.7)
Myocardial infarction	10(11.4)	13(21.7)
Past history of cerebrovascular disease		
Overall	20(7.6)	19(7.1)
Transient ischemic attack	11(55.0)	14(73.7)
Ischemic stroke	7(35.0)	5(26.3)
Carotid angioplasty	2(10.0)	0(0.0)
Carotid endarterectomy	0(0.0)	0(0.0)
Risk factor profile		
*Smoking status*		
None	79(29.9)**	224(84.2)
Former	130(49.2)	18(6.8)
Active	55(20.8)	24(9.0)
*Type II DM*	140(53.0)*	175(65.8)
*Hypertension*	237(89.8)	233(87.6)
*Dyslipidemia*	165(62.5)	182(68.4)
Vital signs	mean ± SD	
Systolic blood pressure (mm/Hg)	141.4 ± 21.5	146.7 ± 21.5
Diastolic blood pressure (mm/Hg)	85.4 ± 12.1	87.4 ± 11.6
Pulse (beat/min)	80.5 ± 9.5	81.5 ± 10.0
Weight (kg)	81.8 ± 12.7	78.3 ± 14.7
Height (cm)	169.9 ± 6.6	158.3 ± 7.0
Waist circumference (cm)	101.5 ± 10.8	102.6 ± 11.8

* p < 0.01 and ** p < 0.001; compared to female patients

A total of 942 antihypertensive drugs were used in the entire study population. The most frequently prescribed antihypertensives were renin-angiotensin system blockers (angiotensin converting enzyme inhibitors [ACEI] and angiotensin receptor blockers [ARB]) (n = 399, 42.4%), followed by diuretics (n = 254, 27%), calcium channel blockers (n = 153, 16.3%), beta-blockers (n = 114, 12%), and alpha-blockers (n = 22, 2.3%). The mostly commonly used antidiabetic drug was metformin (38.8%), the most common lipid lowering drug was atorvastatin (69.5%), and the most common antithrombotic drug was acetyl salicylic acid (ASA) (83.3%).

### PAD symptoms and findings, and ankle brachial index measurements

The three-item questionnaire on PAD symptoms indicated that 42.9% of patients had pain or discomfort while walking and that 25.1% had pain or discomfort while sitting. A total of 35.3% reported relief from pain following 10 min of rest. Physical examinations indicated absence of pedal pulse in 6.5% of patients, arterial murmur in 6.6% of patients, and trophic foot changes in 17.6% of patients.

An abnormal ABI (≤ 0.90) was present in 20.0% of the participants, with no difference between males and females (21.2% for men *vs. *18.2% for women). The frequency of low ABI (≤0.90) was higher for older patients, and almost exceeded 30% for patients more than 70 years old. Advanced age, higher number of risk factors, arterial murmur, trophic changes in the foot, and PAD symptoms were associated with increased likelihood of low ABI (*p *< 0.001; Table 2). The prevalence of low ABI was 35.8% (n = 62) in the 173 patients with histories of cardiovascular events and was 19.1% in patients with intact low extremity pulses. Among patients who had an absent ankle pulse, 67.6% had an ABI greater than 0.90. A total of 58.2% (53 of 91) of patients with trophic foot changes had ABIs greater than 0.90. A total of 15.1% of patients with low ABI had no trophic foot changes.

**Table 2:** Ankle Brachial Pressure Index (ABI) in terms of demographics, clinical features and risk factors

	**ABI ≤0.90** **(n = 106)**	**ABI >0.90** **(n = 424)**
***Gender***	**n(%)**	
Males	56(52.8)^a^	208(49.1)
Females	50(47.2)^b^	216(50.9)
***Age groups***		
50-59 years	20(18.9)^*+*^	182(42.9)
60-69 years	49(46.2)	156(36.8)
70-79 years	37(34.9)^*+*^	86(20.3)
***Presence of risk factors***		
Only 1 risk factor	5(4.7)^*q*^	53(12.5)
2 risk factors	29(27.4)	157(37.0)
3 risk factor	53(50.0)^*q*^	162(38.2)
4 risk factor	19(17.9)	52(12.3)
***Past history of atherothrombotic event***	62(58.5)^c^	111(26.2)
***PAD symptoms***		
*Pain/discomfort while walking*	75(70.8)**	155(36.6)
*Pain/discomfort while standing or sitting*	15(14.2)*	116(27.4)
*Pain/discomfort relieve in <10 minutes*	31(29.2)	117(27.5)
***Positive findings on physical examination***		
*Pedal pulse*	94(88.8)	397(93.4)
*Arterial murmur*	17(16.0)**	16(3.8)
*Trophic changes of the foot*	38(35.8)**	53(12.5)

95% CI : ^a^16.28 - 26.14; ^b^14.10 - 23.49 and ^c^28.69 - 42.98; ^+^p < 0.001 and ^q^p < 0.05

** p < 0.001 and *p < 0.01; compared to absence of the specific finding

Table 2 shows the frequency of low ABI based on the number of major cardiovascular risk factors. For the entire study population, 53.9% (n = 286) had three or more major cardiovascular risk factors (as defined in our according inclusion criteria) and 25.1% of these patients (n = 72) had ABIs indicative of PAD. Among patients with low ABIs, 67.9% had three or more risk factors. There was a significant association of low ABI and number of risk factors in men (*p *= 0.02) but not in women (*p *= 0.134). Women with low ABIs were more likely to have fewer risk factors (Table 2).

We separately evaluated patients with DM (n = 315) and those without DM (n = 215). In the DM group, the frequency of low ABI was 22.9% (21.7% in women *vs. *24.3% in men, *p *= 0.589); in the non-DM group, the frequency of low ABI was 15.8% (13.2% in women *vs. *17.7% in men, *p *= 0.474).

We also calculated the percentage of patients with abnormally high ABIs (> 1.4). There were 32 participants (6%) who had ABIs greater than 1.4, and high ABI was more common in men (n = 23, 8.7%) than in women (n = 9, 3.4%) (*p *< 0.001). When a lower cut-off value was used (>1.30), 64 participants (12%) had high ABIs, and high ABI was also more common in men (n = 46, 17.4%) than in women (n = 18, 6.7%) (*p *< 0.001).

### Diagnostic and therapeutic recommendations by the physicians

A total of 210 participants (114 patients with ABI ≤ 0.90, 96 patients with ABI >0.90) were recommended for further diagnostic procedure(s). Ordering of these diagnostic procedures was more common in patients with abnormal ABI (0.05 >*p *> 0.001; Table 3). Overall, ultrasonography was the most frequently used procedure (61.4%), regardless of ABI (Table 3).

**Table 3:** Evaluation of Ankle Brachial Pressure Index (ABI) in terms of diagnostic methods and selected treatments

	ABI ≤ 0.90 (n = 106)	ABI >0.90 (n = 424)
***Suggested diagnostic method***	**n(%)**	
Conventional angiography	17(14.9)**	15(15.6)
Computed-tomography angiography	4(3.5)*	3(3.1)
Magnetic resonance angiography	23(20.2)**	10(10.4)
Ultrasonography	70(61.4)**	68(70.8)
***Recommended treatment***		
Exercise	83(78.3)**	95(22.4)
Revascularization	31(29.2) **	4(0.9)
Endovascular interventions	4(3.8)*	2(0.5)
Surgery	2(1.9)	1(0.2)

*p < 0.05 and **p < 0.001; compared to ratio of treatment suggestion for patients having ABI >0.90

Exercise (*p *< 0.001), revascularization (*p *< 0.001), and endovascular interventions (*p *< 0.05) were more commonly recommended for patients with low ABI (Table 3).

Pharmacotherapy was recommended to 317 of 530 patients, and ASA was the most frequently prescribed medication (45.4%), followed by pentoxyphylline (16.4%) and clopidogrel (16.09%). The other prescribed medications were statins, warfarin, diosmine, diosmine plus hesperidine, metformin, nifedipine, dipyridamole, and calcium dobesilate.

### Personal awareness and PAD management by the CAREFUL investigators

We evaluated physician awareness of PAD and its management by an eight-item questionnaire before initiation of the study (Table 4). The results indicated that physicians in internal medicine clinics were more likely to order an ABI measurement for males and for patients with younger ages (note that the minimum age is 55 years in the questionnaire). While they ordered an ABI measurement in about two-thirds of patients with DM and smoking habits, they ordered the test for less than half of obese individuals. Almost 80% of physicians thought that patient history and physical examination findings showed no evidence of PAD, and 95% were aware of the diagnostic success of ABI measurement and Doppler examination. The recommended medication for management of PAD varied, but the majority agreed on the benefits provided by smoking cessation and exercise.

**Table 4:** Answers to PAD questionnaire applied to investigators expressed as n(%) (n = 53)

*In which age group (years) of your patients do you consider to measure ABI in your clinic?*						
	55-59	60-64	65-69	70-74	75-79	≥80

For male patients	40 (75.5)	29 (54.7)	31 (58.5)	21 (39.6)	20 (37.7)	17 (32.1)
For female patients	31 (58.5)	31 (58.5)	32 (60.4)	21 (39.6)	20 (37.7)	17 (32.1)

*Do you evaluate obese patients by physical examination and ABI with the suspect of PAD?*						

Yes	24(45.3)					

*Do you evaluate diabetic patients by physical examination and ABI with the suspect of PAD*						

Yes	35(66.0)					

*Do you evaluate smokers by physical examination and ABI with the suspect of PAD?*						

Yes	33(62.5)					

*What are the objectives of PAD treatment according to Turkish Society of Cardiovascular Surgery?*						

To control symptoms	39(73.6)					
To prevent amputation	45(84.9)					
To increase survival	46(86.8)					

*Do "patient history" and "physical examination" show that your patient has PAD?*						

Yes	12(22.6)					

*What should be the option(s) of your patient for the further investigation of PAD?*						

ABI + Doppler	50(94.3)					
Cardiovascular surgery consultation	16(30.2)					
Nothing, routine follow up	3(5.7)					

*What are the treatments of choice for ischemic events?*						

	Continue		Add		Stop	Contraindicate
ASA	48(92.5)		16(30.2)		0(0.0)	0(0.0)
Clopidogrel	30(56.6)		28(52.8)		0(0.0)	0(0.0)
Cilostazole	9(17)		12(22.6)		0(0.0)	0(0.0)
Statins	41(77.4)		24(45.3)		0(0.0)	0(0.0)
Other lipid lowering drugs	26(49.1)		22(41.5)		1(1.9)	0(0.0)
ACE inhibitors	38(71.7)		21(39.6		0(0.0)	0(0.0)
Other antihypertensives	24(45.3)		24(45.3)		3(5.7)	1(1.9)
Warfarin	23(43.4)		21(39.6)		3(5.7)	0(0.0)
Antihyperglycemic drug	40(75.5)		17(32.1)		0(0.0)	0(0.0)
Stop smoking program	45(85.9)		20(37.7)		1(1.9)	1(1.9)
Walking-exercise prog.	43(81.1)		23(43.4)		1(1.9)	0(0.0)

## Discussion

In our study population, the overall frequency of low ABI (≤ 0.90) was 20.0%, and the frequency of low ABI was similar for males and females. Older age, greater number of risk factors, and presence of PAD-related physical findings were associated with increased likelihood of low ABI.

Previous studies have shown that a low ABI has specificity greater than 98% for the diagnosis of PAD and specificity greater than 92% for the prediction of CHD and stroke [16-18]. The frequency of PAD, as determined by low ABI in adults with no known CVD, varies from 3.7 to 14% in different populations [4,8,15,24-29]. However, for people with established CVD or other measures of atherosclerosis, the prevalence of low ABI was reported as 15% to 40% [6,7,23,30], in accordance with the results of the CAREFUL study. Another significant finding in the present study is that age above 70 years is associated with a 30% prevalence of PAD, independent of the presence of a major risk factor for CVD. This is also in accordance with a previous study with a similar design of a different population [6].

Previous studies have reported different results regarding gender differences in the prevalence of low ABI. In our study, the frequency of low ABI was similar in women and men with established CVD (other than PAD) or cardiovascular risk factors. These results are in line with previous studies with similar designs, although subgroup analysis indicated that disease was more prevalent in men with established CVD [31,32]. The Atherosclerosis Risk in Communities Study (ARIC) cohort study indicated that low ABI was rare in middle-aged and younger people [33]. Likewise, other studies indicated that the prevalence of low ABI increases substantially with age [1,15]. Thus, our results, which indicate an increased prevalence of low ABI in older patients, are compatible with these previous studies.

DM is accepted as a CVD equivalent, but the prevalence of PAD in DM patients ranges from 10% [34] to 20.9% [35], comparable to the ratio reported in reference populations. We found a higher prevalence of PAD in patients with DM relative to previous reports and relative to the average of the general study group. The presence of DM increases the risk of PAD more than four-fold [36], and the American Diabetes Association recommends PAD screening by ABI every five years for individuals with DM, even before the age of 50 years or in the presence multiple risk factors [37].

The prevalence of high ABI was 6% for a cut-off of 1.40 and 12% for a cut-off of 1.30, indicating a similar or a somewhat higher incidence than several previous studies [8,38,39]. Although the mechanism is not clear, there is an established association between elevated ABI and higher mortality [8]. One possible explanation is that elevated ABI may indicate the presence of generalized arterial stiffness [40], which places an individual at high or very high risk, even in the absence of other risk factors [41].

A previous longitudinal study, which showed that symptomatic and asymptomatic subjects with PAD had higher cardiovascular mortality than subjects without PAD, indicates the prognostic importance of asymptomatic PAD [10]. Previous studies reported a prevalence of PAD-related symptoms (mainly intermittent claudication) from 5.3% to 18.9% [7]. This low prevalence was interpreted as being due to elderly people not walking enough to experience symptoms because of impaired vascularization of the extremities or other disorders, such as osteoarthritis. In contrast, the prevalence of PAD symptoms was 35% in our population of patients, who were all more than 50 years old. However, although a previous study reported that women with PAD were less likely than men to report symptoms of intermittent claudication [7], we found no such difference in the present study. In fact, such differences in demographic distributions of screened populations or analysis of populations with higher risk for PAD were indicated to be the causes of the differences in the prevalence estimates of published studies [42,43].

Signs and symptoms may be inadequate for the diagnosis of PAD, so previous researchers have proposed the use of non-invasive testing when history and vascular examination yield ambiguous results [31]. In this context, a previous study illustrated the difficulty in using clinical symptoms to diagnose PAD, because 47% of people with PAD-related symptoms had normal ABIs [11]. Accordingly, symptoms suggested a diagnosis of PAD in 35% of our population, and positive findings indicating PAD were evident in the physical examination in only 6-17% of our patients, whereas abnormal ABIs confirmed PAD diagnosis in 20% of our population. In fact, ABI is considered most beneficial in identifying asymptomatic individuals, rather than those with preexisting clinical disease, who are already targeted for intervention [15]. Furthermore, we found that the likelihood of having low ABI was higher in the presence of PAD-related symptoms. In support of this finding, a previous study found that most atherosclerotic events occurred in symptomatic patients with low ABIs [26].

The CAREFUL study indicated that arterial murmur or trophic changes were associated with the presence of low ABI. However, the prevalence of low ABI in participants with intact low extremity pulses on physical examination was 19.1%, similar to the percentage in the whole study population. It is known that the absence of low extremity pulse is indicative of PAD, but the presence of low extremity pulses is not sufficient to rule out PAD [20]. In our study, patients without low extremity pulses had almost a two-fold higher prevalence of PAD. Thus, the absence of lower extremity pulse is insufficient for a firm diagnosis of PAD.

An additional goal of the present study was to assess physician awareness of PAD. In particular, we considered physicians' identification of patient risk, symptoms and clinical findings, diagnostic procedures, and patient management. We noted that diagnostic and follow-up procedures selected by the CAREFUL investigators were comparable to management recommendations in the guidelines [20]. Physicians who participated in our study indicated that they evaluated subjects with DM, smokers, and obese individuals by physical examination and ABI based on the suspicion of PAD. Based on Turkish Society of Cardiovascular Surgery guidelines, the main objectives of PAD treatment are to increase patient survival, prevent amputation, and control symptoms. On the other hand, almost half of physicians studied here reported that they had no clear idea of the role of patient history and physical examination in the diagnosis of PAD. This may not be surprising, because clinical examination alone does not allow confirmation or exclusion of PAD and cannot be used for definitive clinical decision-making [44].

In line with the latest guidelines [20,45], pharmacotherapy was recommended for more than half of our study population. Likewise, the majority of our physicians recommended ASA. Although the overall rate of pharmacotherapy administration in our study (59.8%) was better than that reported in the REACH registry (28.7%), use of antiplatelet therapy in our patients (45.43%) was much lower than in the REACH registry (81.7%) [9]. Furthermore, although not reflected in real clinical practice, half of our investigators identified that addition of clopidogrel to their treatments could be a therapeutic option for ischemic events. For this reason, as indicated in the literature [46], it seems necessary to improve adherence to PAD treatment guidelines, as evidence suggests this would improve patient outcome. Our physicians had excellent awareness of the benefits of exercise, particularly for people with low ABIs, and this was recommended by over 80% of physicians. On the other hand, despite the strong evidence concerning available recommendations, recent data from the REACH registry indicate a significant gap between treatment recommendations for PAD and actual clinical practice [9].

The main limitations of the present study were the questionable representativeness of our study population (ecological fallacy), the cross-sectional study design, and the lack of blood sample analysis. Nevertheless, our findings suggest that measurement of ABI has the potential to improve estimation of cardiovascular risk because it is a simple, inexpensive, and rapid measurement tool, and can detect the presence of peripheral atherothrombosis in patients with current or prior history of other vascular disease and, more importantly, in at-risk patients.

## Conclusions

In conclusion, we found a significantly higher prevalence of low ABI in older individuals, those with more total risk factors, and those with PAD symptoms. The present study emphasizes the diagnostic and prognostic implications of low ABI in high-risk subjects, and the results are comparable to similar registries of different populations.

## Competing interests

The authors declare that they have no competing interests.

## Authors' contributions

AKB, IT and YK conceived of the study, and participated in its design and coordination and helped to draft the manuscript. OT and MG drafted the manuscript. All authors read and approved the final manuscript.

## Pre-publication history

The pre-publication history for this paper can be accessed here:

http://www.biomedcentral.com/1471-2261/11/4/prepub
